# Snow avalanches are a primary climate-linked driver of mountain ungulate populations

**DOI:** 10.1038/s42003-024-06073-0

**Published:** 2024-04-29

**Authors:** Kevin S. White, Eran Hood, Gabriel J. Wolken, Erich H. Peitzsch, Yves Bühler, Katreen Wikstrom Jones, Chris T. Darimont

**Affiliations:** 1 Department of Natural Sciences, Program on the Environment, University of Alaska Southeast, Juneau, AK 99801 USA; 2 Department of Geography, University of Victoria, Victoria, BC V8W 2Y2 Canada; 3 Division of Wildlife Conservation (ret.), Alaska Department of Fish and Game, Juneau, AK 99811 USA; 4 Alaska Division of Geological and Geophysical Surveys, Climate and Cryosphere Hazards Program, Fairbanks, AK 99709 USA; 5 Alaska Climate Adaptation Science Center, University of Alaska Fairbanks, Fairbanks, AK 99775 USA; 6 U.S. Geological Survey, Northern Rocky Mountain Science Center, West Glacier, Montana, MT 59936 USA; 7 WSL Institute for Snow and Avalanche Research SLF, Davos CH-7260, Davos, Switzerland; 8 Climate Change, Extremes and Natural Hazards in Alpine Regions Research Centre CERC, Davos CH-7260, Davos, Switzerland

**Keywords:** Population dynamics, Climate-change ecology, Conservation biology, Animal behaviour

## Abstract

Snow is a major, climate-sensitive feature of the Earth’s surface and catalyst of fundamentally important ecosystem processes. Understanding how snow influences sentinel species in rapidly changing mountain ecosystems is particularly critical. Whereas effects of snow on food availability, energy expenditure, and predation are well documented, we report how avalanches exert major impacts on an ecologically significant mountain ungulate - the coastal Alaskan mountain goat (*Oreamnos americanus*). Using long-term GPS data and field observations across four populations (421 individuals over 17 years), we show that avalanches caused 23−65% of all mortality, depending on area. Deaths varied seasonally and were directly linked to spatial movement patterns and avalanche terrain use. Population-level avalanche mortality, 61% of which comprised reproductively important prime-aged individuals, averaged 8% annually and exceeded 22% when avalanche conditions were severe. Our findings reveal a widespread but previously undescribed pathway by which snow can elicit major population-level impacts and shape demographic characteristics of slow-growing populations of mountain-adapted animals.

## Abstract (web summary)

Snow avalanches—a direct physical process—represent a major source of mortality and comprise a previously undescribed pathway by which climate-linked mechanisms impact demographics of slow-growing populations of mountain-adapted animals.

## Introduction

Climate change is occurring rapidly in mountain environments^1,2^, imposing profound changes to sensitive ecological communities and processes. Multiple and novel stressors can harm species such as alpine ungulates, which have specialized adaptations and narrow biophysical niches^3–5^. Questions remain, however, about potential demographic implications and their underlying mechanistic drivers^5,6^. Seasonal snow conditions might play a central role, and can act as a primary influence on ungulate population dynamics^7,8^. Identified mechanisms are largely ecological and physiological, with changes in snow depth and distribution altering energetic costs of locomotion, vulnerability to predation, and accessibility and quality of forage in both summer and winter^9–12^. We take a different focus, showing here how snow avalanches act as a direct, physical process that cause high levels of mortality and strongly influence demography in mountain wildlife populations.

Mountain ungulates are behaviorally predisposed and morphologically adapted to steep, rugged terrain to avoid the risk of predation (Supplementary Fig. 1)^13^. Such specialization, however, may carry other risks. Specifically, slopes that provide effective refugia from predators are also subject to frequent avalanching. Indeed, avalanche mortalities have been described for several ungulate species^14–17^. However, the difficulties associated with systematically documenting avalanche fatalities, which requires marking and long-term monitoring of individuals across broad geographies in dangerous mountain conditions, have precluded a definitive demographic assessment. To address this gap, we combined an extensive, individual-based mountain goat field monitoring data set with spatially explicit avalanche terrain data to quantify how avalanches influence the population ecology of mountain wildlife.

**Fig. 1:**
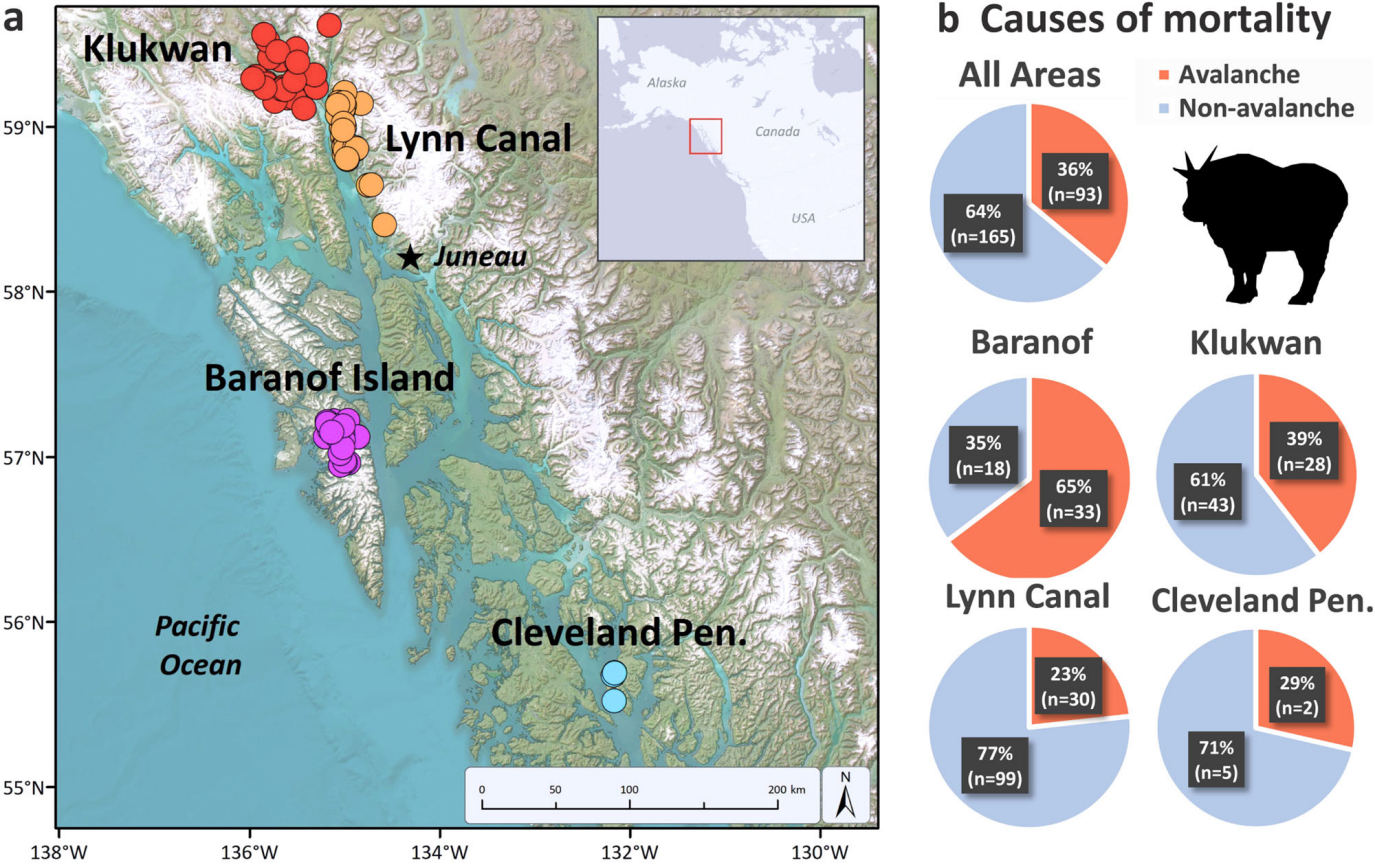
Avalanche mortality across populations. **a** Map depicting locations where radio-marked mountain goats died in avalanches during 2005−2022 in four study areas in southeastern Alaska. **b** The proportion of collared mountain goats that died from avalanches (*n* = 93) vs. non-avalanche related causes (*n* = 165) by study area and summarized across the region.

We collected data on mountain goats and their environment in southeastern Alaska, USA. The area’s Coast Mountains are characterized by steep, rugged topography, with avalanche activity observed across the full vertical range of habitat occupied by mountain goats (0 to >1500 m). To quantify mountain goat exposure to and mortality from avalanches, we affixed global positioning system (GPS) and very high frequency (VHF) radio-collars to 421 animals from four populations over a 17-year period (*n* = 1218 mountain goat years). The four populations inhabit a ~ 500 km domain characterized by a broad range of biogeographic settings (Fig. 1a).

## Results

### Avalanche mortality in mountain goats

To determine cause of mortality, we intensively monitored survival status and identified the timing and location of mortality events. We found that avalanches comprise a major source of mortality, accounting for 23 to 65% (mean = 36%; *n* = 93) of average annual mortality, depending on population (*n* = 258; Fig. 1b). Avalanches were a more common cause of mortality for females (41%, *n* = 39) compared with males (33%, *n* = 54). These mortalities predominantly (61%) comprised prime-aged (4−9 yrs old) individuals for both females (54%) and males (67%), age classes that otherwise have the highest survival rates and reproductive contribution (Supplementary Figs. 2 and 3)^5,15,18^. Avalanche mortality varied spatially and temporally across populations in relation to geographic, climatic, and ecological characteristics of regional study areas, being highest on Baranof Island (65%, *n* = 51), relative to Klukwan (39%, *n* = 71), Cleveland Peninsula (29%, *n* = 7), and Lynn Canal (23%, *n* = 129; Fig. 1b). Avalanche mortalities occurred across nine months of the year, and peaked when snow conditions were most unstable during early season snowpack development (October and November) and the spring melting period (April and May; Supplementary Fig. 4).

The high levels of mortality highlight the challenges mountain ungulates face in mitigating avalanche risk. Avalanche formation involves the interaction among meteorological conditions, snowpack, and terrain. Exposure to avalanche hazard depends largely on topography and the prevalence of structural weaknesses in the snowpack that vary in space and time^19^. While we did not explicitly test whether mountain goats select specific terrain types to avoid avalanches during risky periods, the complex and dynamic physical interactions that create avalanche vulnerability are likely difficult to detect among wildlife, minimizing opportunity for development of behavioral strategies to avoid avalanche hazards in areas and periods of snowpack instability. Thus, compared to mortality mechanisms such as predation (for which most attempts end in prey escape) and winter starvation that can be mitigated by learning and behavioral adaptations, avalanches may represent a ‘wicked problem’; that is, there are limited opportunities for trial-and-error learning due to the catastrophic outcomes that follow initial exposure^20,21^. By extension, opportunities for and pace of fine-scale behavioral adaptation may be constrained because risk perception of such cryptic and stochastic processes is likely weak and not strongly linked to heritable variation of behavioral responses^22^.

### Costs of living dangerously

**Fig. 2:**
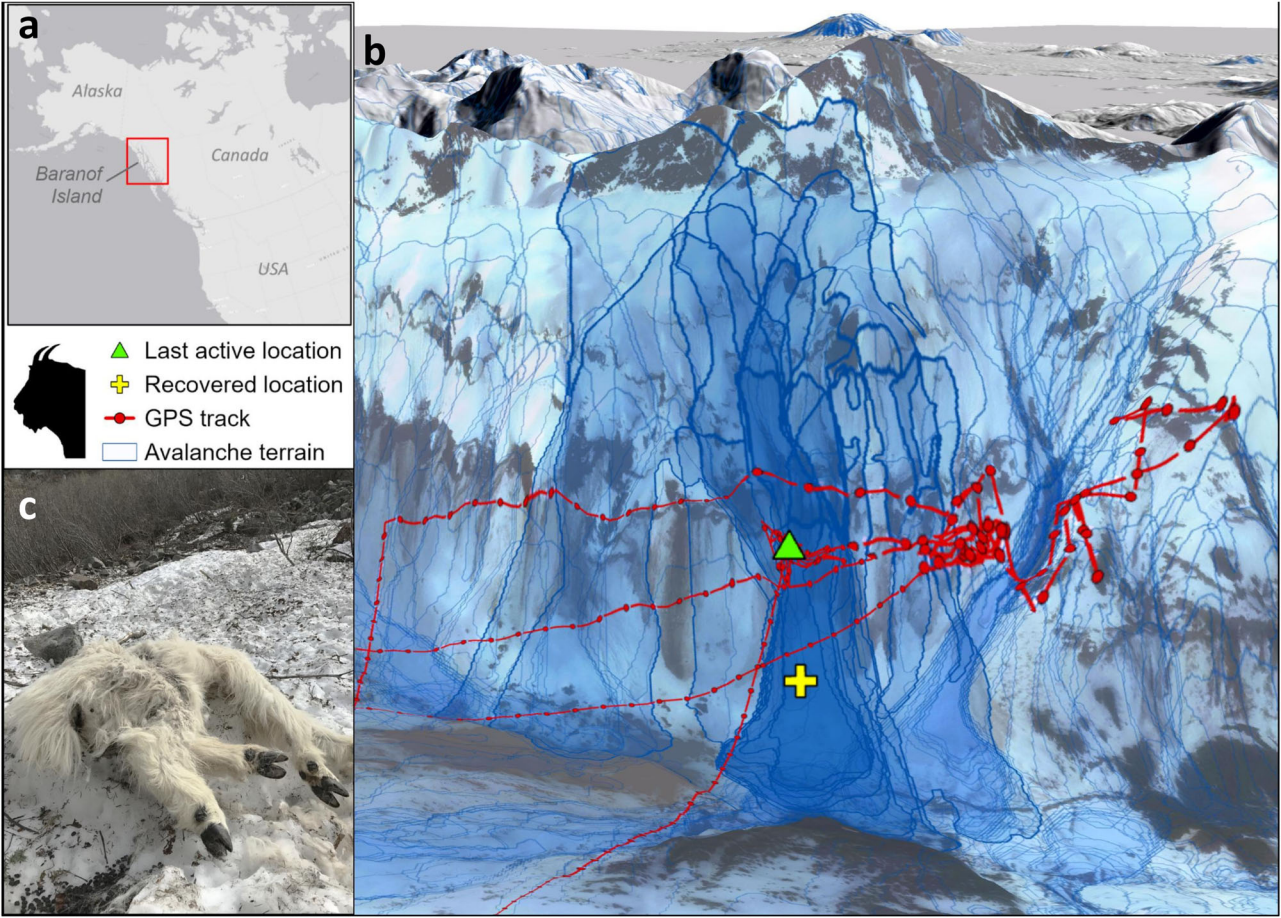
Estimation of mountain goat use of avalanche terrain. **a** Mountain goat study location where (**b**) GPS radio-collar location data were intersected with large-scale avalanche hazard indication maps to quantify individual mountain goat exposure to avalanches. The example 3D image illustrates typical winter use of avalanche terrain by an adult female mountain goat (BG041) that was caught (triangle) and subsequently buried (cross) by an avalanche in the Baranof Island study area in January 2016. Simulated avalanches that swept through the mountain goat mortality site are delineated (thick blue outlines), as well as all other avalanches in the area (light blue outlines). **c** A mountain goat (KG034) carcass partially buried in avalanche debris, near Klukwan, Alaska in May 2017. The animal suffered a depressed skull fracture among other critical injuries.

We hypothesized that putatively stochastic avalanche mortality events would be linked, in aggregate and across populations, with the amount of time mountain goats spend in avalanche terrain during months with snow cover. Accordingly, we modeled the potential release area locations^23^ and the maximum spatial extent of simulated individual avalanches within the geographic range of the study populations using Rapid Mass Movement Simulation (RAMMS), a numerical dynamic avalanche simulation model^24^. Delineating avalanche hazard zones allowed us to quantify prevalence and use of avalanche terrain in winter for individual mountain goats, assess the physical setting of avalanche mortality sites, and, in some cases, track the precise locations of avalanche entrainment and burial of killed individuals (Fig. 2). We then evaluated whether exposure to avalanche hazard varied seasonally by intersecting temporally referenced GPS radio-collar locations (*n* = 801,410 locations, 367 individuals) with the avalanche hazard spatial data layer (Fig. 2).

**Fig. 3:**
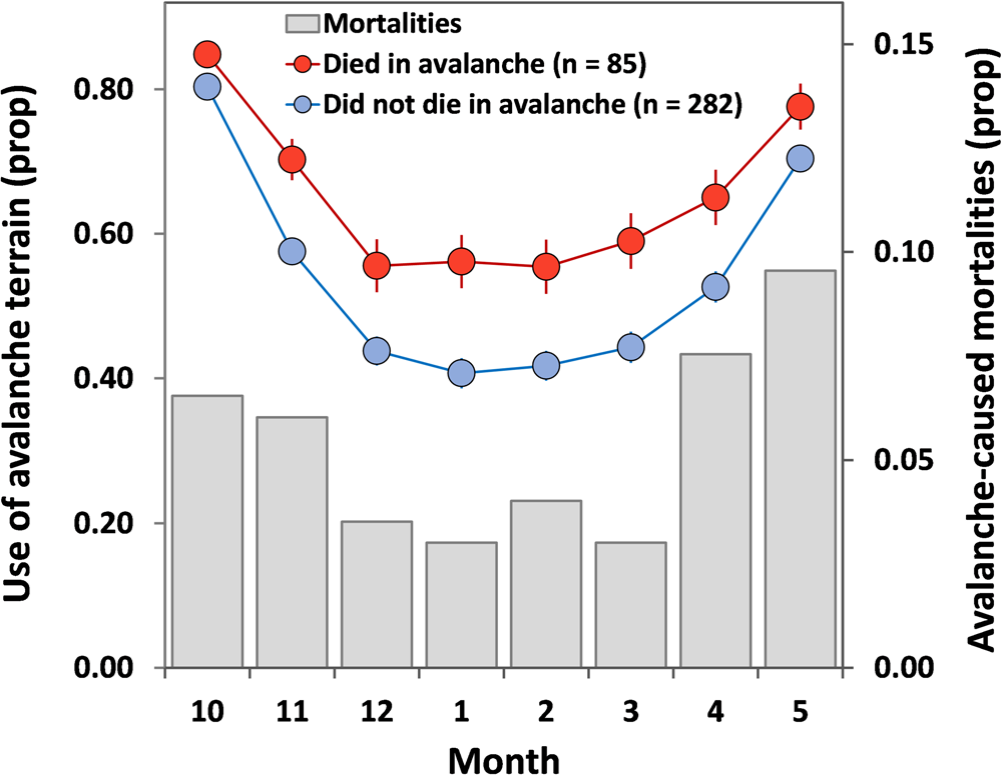
Mountain goat use of avalanche terrain and survival. GPS radio-collared mountain goats that died in avalanches (red circles, *n* = 85) used significantly more avalanche terrain each month relative to individuals that did not die in avalanches (blue circles, *n* = 282; *P* < 0.05 for all months). Error bars represent 95% confidence intervals. Proportion of mortalities caused by avalanches is summarized by month for the months with snow (gray bars, secondary y-axis) and includes all GPS plus VHF radio-monitored animals (total avalanche mortalities, *n* = 93). Data were collected in four study areas in southeastern Alaska during 2005−2022. GPS radio-collar location data was collected from a subset (87%) of the total individuals monitored (*n* = 421). Avalanche terrain includes non-forested predicted avalanche release areas and avalanche paths derived using the RAMMS avalanche simulation model.

Mountain goat use of avalanche terrain was widespread and linked to mortality. Avalanche release areas and paths constitute most (62%) of the alpine and subalpine footprint across the winter range. There was little variability in the proportion of avalanche terrain in the three largest study areas, which ranged from 57% in Lynn Canal to 67% in Klukwan; however, in the smallest study area, Cleveland Peninsula, avalanche terrain comprised only 17% of the wintering area (Supplementary Table 1). Across all months and populations, mountain goats that died in avalanches exhibited significantly higher use of avalanche terrain (67 ± 3%, *n* = 85) than those that did not die in avalanches (54 ± 2%, *n* = 282, *t* = 3.643, *P* < 0.01), a pattern that we likewise observed in finer-scale temporal analyses (i.e., for each individual month during the snow period; Fig. 3 and Supplementary Table 2). Use of avalanche terrain varied substantially among populations, helping to explain observed spatial differences in avalanche mortality. Lynn Canal and Cleveland Peninsula, where animals used avalanche terrain less than 40% of the time in winter months, had the lowest proportion of avalanche mortalities. The Klukwan and Baranof populations, where animals occupied avalanche terrain more than 70% of the time in winter, had avalanche mortality rates roughly double those of the other areas (Fig. 1b and Supplementary Fig. 5).

### A new view of snow and mountain ungulate population dynamics

**Fig. 4:**
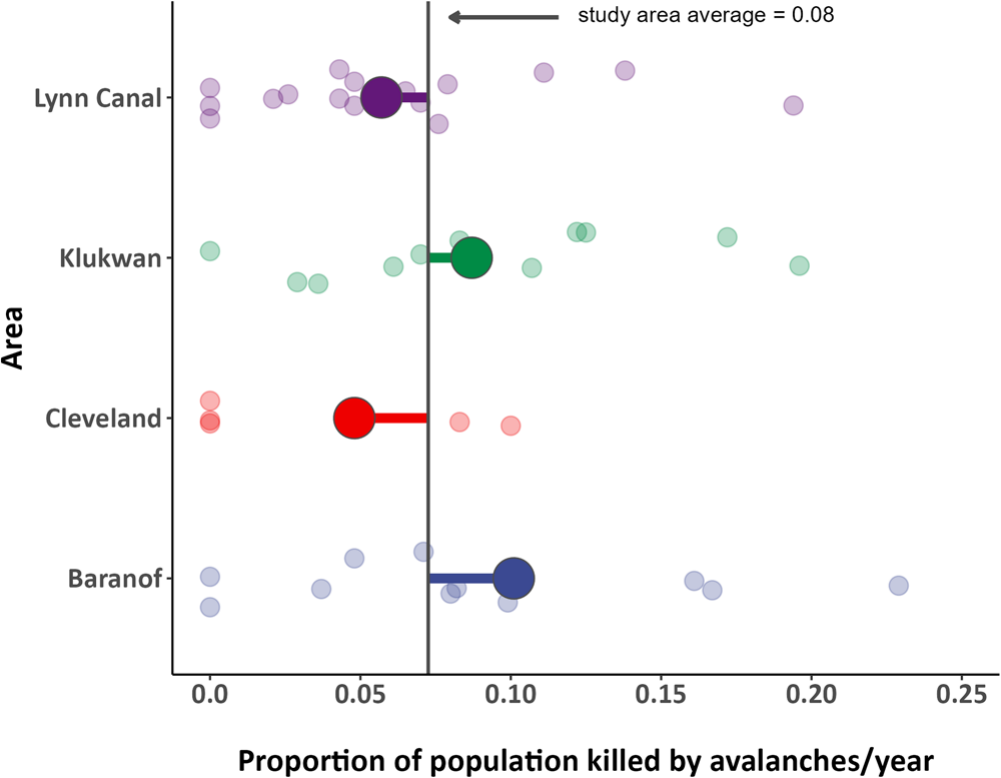
Spatiotemporal variation among populations in avalanche-caused mortality. Proportion of radio-marked mountain goats that died due to avalanches in a given year for each southeastern Alaska study area during 2005−2022. Average estimates are depicted by the large colored circles, and small circles represent annual study area estimates. The black vertical line delineates the average across all four study areas and years.

Avalanche mortality patterns scaled up to reveal population-level implications. Estimated from radio-marked individuals, the proportion of the population that died from avalanches averaged 8% annually over the study (*n* = 43 population-years) and showed substantial spatial and temporal variation (Fig. 4). Three populations had at least one year where more than 15% of the population died in avalanches, with peak annual avalanche mortality of over 22% of the Baranof population. Yet, we also documented years without avalanche mortalities in all four populations. Such variation suggests a complex relationship between snow and ungulate population ecology. In particular, the prevailing ecologically-focused view that snow depth and coverage is the primary snow-related control on ungulate fitness^7^ may not hold true for populations exposed to substantial avalanche hazard. Instead, intra-seasonal variability in winter weather, which controls the amount of snowfall and the formation of weak layers in the snowpack^19^, may serve as a key physical driver of population-level mortality.

That population-level mortality from avalanches can exceed 20% in a single year highlights the role of stochastic environmental processes in the viability of inherently vulnerable alpine wildlife. Stochastic predation events in mountain bighorn sheep, for example, can precipitate acute population declines resulting in demographic restructuring and long recovery times^25^. Avalanches may have similar implications for mountain goats. Growth rates of mountain goat populations are particularly low. For example, modeling from this and other areas—that has not incorporated the higher end of variation in annual mortality reported here—suggests that populations are able to sustain only limited annual removals such as by harvest (1−4% annually)^26–28^. In this context, avalanche-driven mortality, which is dominated by prime aged individuals (Supplementary Fig. 2), is capable of eliciting major demographic impacts and may underlie previously documented population declines and extirpation events in fundamentally tenuous mountain ungulate populations^26,28–31^.

## Discussion

**Fig. 5:**
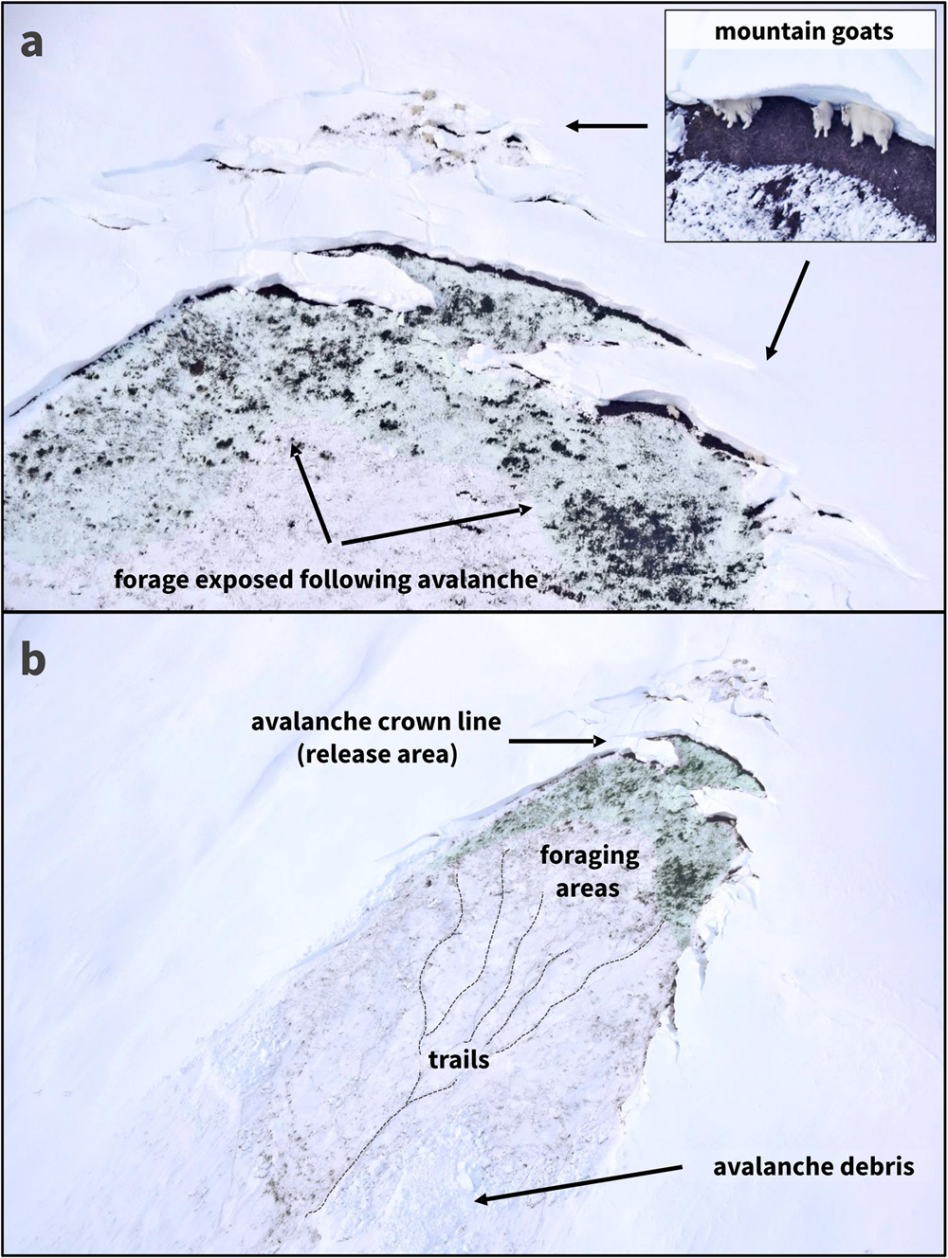
Avalanches and winter habitat use. **a** Photograph depicting mountain goat foraging following a glide avalanche, Summit Creek, Klukwan, Alaska, February 2021. Glide avalanches extend across the full depth of the snowpack and scour the landscape to expose underlying vegetation (primary foraging area highlighted in light green). Here, two groups of mountain goats used separate locations along the fractured crown line (black arrows). **b** Extensive tracks, visible in the image, indicate foraging activity on the newly exposed, vegetated slope.

Understanding mountain goat use of dangerous, avalanche-prone terrain requires broader consideration of how avalanches might influence multiple components of fitness. Mountain goats utilize steep terrain to mitigate predation-risk, with optimally selected slope angles (36−58°)^32^ closely corresponding with the most avalanche prone slopes (30−45°)^33^ (Supplementary Fig. 1). Additionally, scouring by avalanches provides nutritional benefits by generating and maintaining accessibility of forage rich, early-successional habitats during winter and spring (Fig. 5)^34,35^. Yet, given sufficient exposure, avalanche terrain might manifest as a form of ecological trap. Ecological traps have traditionally been described in contexts where rapid and direct human-induced landscape change (i.e., habitat modification) results in ecological and evolutionary mismatch such that animals, in apparent error, select certain habitats associated with low fitness^36^. Avalanches may thus represent a novel form of ecological trap that is linked to similarly imperceptible seasonal changes in the structure of snow cover blanketing mountain environments. Whether and how climate change might lurk behind the pronounced avalanche-caused mortality we observed is unknown. If mountain goats evolved with similar snow conditions, the benefits of using avalanche-prone terrain must be extraordinarily high for populations to offset such high mortality (in proportion and magnitude).

Population-level variability in avalanche mortality highlights the role of migratory and wintering strategies in exposing mountain ungulates to avalanche hazard. Mountain goats in Lynn Canal are highly migratory and primarily use low elevation forested habitat during winter months, while individuals in Klukwan and Baranof employ mixed-migration strategies, often remaining at higher-elevation during winter to forage in subalpine habitats and on wind-scoured alpine ridges (Supplementary Fig. 5, “Methods – Study System”). Other mountain ungulate species exhibit comparable variation in partially migratory behavior, with a fraction of individuals residing year-round at high elevation while others migrate to low-elevation ranges during winter^37,38^. Partial migration is taxonomically widespread, especially among ungulates^39^. Accordingly, our findings have broad implications, given that selection imposed by avalanches may reduce the prevalence of risk-prone higher-elevation resident strategies. Over time, climate-driven variation in avalanche hazard^40^ may alter fitness trade-offs among migratory phenotypes and, ultimately, the occurrence of partial migration in mountain systems.

Regardless of details yet unknown, climate change impacts on snow characteristics will loom large in the future of mountain ungulates. It will shift the spatial and temporal occurrence of avalanches^41,42^, with implications for exposure and entrapment. Warming will intensify extreme precipitation during winter^43^ and increase the occurrence of rain-on-snow events^44,45^, both of which contribute to snowpack instability and avalanche release^46^. Avalanche character will also shift from dry-snow dominated to wet slides^41^, with potentially increased avalanche mortality rates^47^. At the same time, future increases in snowline elevation in mountain environments may decrease avalanche hazard at lower altitudes^42^. Yet, the demographic influence of avalanches on mountain ungulate populations is likely to persist into the future because both avalanche hazard^42^ and mountain ungulate ranges^5,48^ are expected to shift upward in elevation as climate warms.

The high rates of avalanche mortality we document might be widespread among mountain wildlife, and if so carry important cultural and ecological implications. Mountain environments with avalanche hazard currently cover about 6% of Earth’s land area and occur on all continents^49^, with 32 mountain ungulate species across 70 countries inhabiting a substantial fraction of this range^50^. Ungulate carcasses provide critical nutritional benefits to a diversity of avian and mammalian scavenging specialists (Supplementary Fig. 6)^51^, and are particularly important in mountain food webs characterized by low ungulate biomass^52^. Moreover, Indigenous hunters have relied on mountain ungulate populations for millennia, a relationship involving important subsistence and cultural traditions including use of wool for weaving ceremonial robes and other regalia^53–55^. Mountain ungulates are also highly regarded among contemporary sport hunters and recreational wildlife-viewers worldwide. Thus, recognition that the persistence of ecologically and culturally important mountain ungulate populations relates to climate-linked phenomena in more diverse ways than previously acknowledged has far-reaching conservation and cultural implications for mountain ecosystems and people.

## Methods

### Study system

Mountain goats were studied in four separate areas across a broad geographic range in coastal Alaska (5537 km^2^; Fig. 1 and Supplementary Table 1) from 2005 to 2022. This area is within the Coast Mountains biogeographic region^56^. Mean monthly temperatures range from −2 to 14 °C and mean annual precipitation is 1400 mm in Juneau^57^, the area’s most populous city. Across the region, annual precipitation ranges from 1 to >8 m and winter snowfall ranges from 0.5 to >3 m of snow water equivalent^58^. During the study period, annual snowfall at sea level in Juneau averaged 233 cm with a range of 89−501 cm.

The region is part of the world’s largest contiguous coastal temperate rainforest and composed primarily of Sitka spruce-western hemlock (*Picea sitchensis-Tsuga heterophylla*) forests at lower elevations (below 450−750 m). At higher elevations, subalpine and alpine habitats dominated by krummholtz forest, low-growing herbaceous meadows and ericaceous heathlands are widespread and persist to elevations of about 1400 m. The geologic terrain is complex and strongly influenced by terrain accretion and uplift processes^59^. The resulting landscape is highly fractured and dominated by steep, rugged topography that is fragmented by active glaciers, icefields, high-volume river systems and marine waters^59^. The avalanche paths in this study extend from sea level to 2000 m and include a variety of aspects as a result of the complex topography of the Coast Mountains.

Mountain goats in this region are widespread and occur at low to moderate densities, typical of northern coastal areas inhabited by the species^55,60^. Populations exhibit a high degree of local-scale population genetic differentiation, with limited movement among geographically discrete mountain complexes^28,61,62^. Mountain goats are habitat specialists and select steep, rugged terrain in close proximity to cliffs and exhibit seasonal variation in altitudinal distribution^32,62,63^. Mountain goats are partially migratory, with some individuals, depending on study area, residing in alpine and subalpine habitats throughout the year^64,65^. However, most individuals conduct short-distance (5−10 km), seasonal migrations involving annual movements between high-elevation alpine summer habitats and forested, low-elevation wintering areas^63–65^. Downslope migrations tend to correspond with the first major snowfall events at high elevation (i.e., mid-October), while upslope migrations are timed with onset of the spring snow ablation and pre-parturition period (i.e., early-May)^63^. Individuals in Lynn Canal are highly migratory and, like mountain goats on the Cleveland Peninsula, primarily use low elevation forested habitat during winter months, while individuals in Klukwan and Baranof more frequently employ mixed-migration strategies, more often utilizing higher-elevation subalpine and alpine habitats where avalanche exposure is greater^63,64,66^(Supplementary Fig. 5). Impacts of human development and activity in the study area are, generally, minimal. Nonetheless, low-intensity or localized activities do occur and include regulated hunting, ground- and air-based recreational tourism, timber harvest and mining^28,32,64^. The large mammal predator-prey communities in this area are intact and, in addition to mountain goats, key species include: moose (*Alces alces*), Sitka black-tailed deer (*Odocoileus hemionus sitkensis*), wolves (*Canis lupus*), coyotes (*Canis latrans*), black bears (*Ursus americanus*), brown bears (*Ursus arctos*) and wolverines (*Gulo gulo*); though local variation occurs relative to species distribution and abundance^63,67^.

### Mountain goat monitoring

Adult male and female mountain goats were captured using standard helicopter darting techniques^68^. During handling all animals were fitted with mortality-sensing VHF and/or GPS radio-collars (Telonics Inc., Mesa, AZ). GPS radio-collars were programmed to acquire a GPS location at 6-h intervals; ancillary activity sensor and temperature measurements were collected over a 15-min evaluation period commencing at the initiation of the GPS location acquisition attempt. Age of animals was determined by counting horn annuli^69,70^ and, in some cases, cross validated by examination of tooth eruption patterns (for young animals)^70^ and/or cementum analysis of incisors (for deceased animals; Matson’s Laboratory, Milltown, MT). Capture and handling procedures complied with all relevant ethical regulations for animal use and were approved by the Alaska Department of Fish and Game Institutional Animal Care and Use Committee (protocols 05‐11, 2016‐25, 0078‐2018‐68, 0039‐2017‐39) and followed American Society of Mammalogists guidelines^71^.

Following capture, animals were typically monitored at least once per month (often multiple times per month) via aerial telemetry to determine whether animals were alive or dead. Survival status was also determined via examination of GPS radio-collar location, activity and temperature sensor data, an approach that often enabled temporal determination of death to within a 6-h time window. In cases where animals were determined to have died, an initial fixed-wing aerial reconnaissance of the site was conducted and followed up with a ground-based examination to determine context and causes of death, to the extent possible. Due to safety and logistic considerations, ground-based examinations were typically conducted after initial aerial reconnaissance and determination of death. Due to the delay, it was not always possible to definitively distinguish between non-avalanche related causes of death (i.e., due to scavenging of carcasses). However, avalanche-caused mortality determinations were definitive and associated with carcasses being buried under, or associated with, avalanche debris and located within active avalanche paths.

### Avalanche simulations and mapping

Avalanche hazard indication maps were developed from terrain analysis, downscaled climate model reanalysis, and numerical simulations of avalanche runout dynamics. Object-based image and terrain analyses were used with a digital terrain model (DTM; 5-m resolution) to determine avalanche potential release areas outside of closed canopy, conifer forest areas^23,72^. Dynamically downscaled climate reanalysis (4-km resolution)^73^ was used to calculate the maximum snow depth increase over three days in the 1981−2010 climatology, which was used to determine the avalanche release depth for each potential release area. We recognize that biologically meaningful avalanche activity can occur within closed-canopy forests but maintain that such events are very uncommon in southeast Alaska relative to avalanche activity in alpine areas. As such, for this large-scale approach we assumed that closed canopy, conifer forest areas were not prone to significant avalanche activity and restricted our automated mapping of potential release areas to landcover types outside this designation. Potential release areas and release depths were then used in the numerical dynamic avalanche model RAMMS^24^ to simulate millions of individual avalanches within the study areas and map avalanche hazard following the large scale hazard indication modeling approach developed by ref. ^74^. Mapped avalanche hazard zones were further used to confirm that all mortalities classified as avalanche-related were located in avalanche hazard zones.

### Mountain goat spatial analyses

Mountain goat GPS radio-collar location data were compiled and subsequently filtered, using methods described by refs. ^75,76^, to ensure geolocational accuracy. Using a geographical information system, mountain goat GPS location data were intersected with avalanche hazard indication maps to determine relative proportion of time each individual mountain goat spent in avalanche terrain during months when avalanche mortalities occurred (Oct−May). We defined avalanche terrain as avalanche potential release areas and runout paths combined, as both features comprised equivalent risk to mountain goats. Proportional use of avalanche terrain was calculated for each individual and coded based on whether the individual did or did not die in an avalanche. Monthly and seasonal differences in proportional use avalanche terrain was analyzed in relation to fate using paired students *t* tests, with *P* < 0.05 denoting statistical significance.

### Mountain goat mortality and survival estimation

As described above, causes of mortality were ascertained for every deceased individual. All causes of mortality were summarized as either being caused by an avalanche or other, non-avalanche related cause(s), including unknown (Supplementary Table 3). Cause-specific mortality was summarized for each population across all years of study as well as by month and study area. Survival of radio-collared animals was calculated for the annual cycle (June−May), at monthly time steps, using the Kaplan−Meier estimator^77^. This method allows for staggered entry and exit of newly captured or deceased animals, respectively. While post-capture effects were not evident in our study, we implemented a conservative approach and excluded mountain goats for survival analysis for three days after capture (following ref. ^78^). Survival was estimated using only avalanche-caused mortality cases in order to determine the proportion of radio-marked animals that died due to avalanches (i.e., population-level mortality) for each year and study area. To ensure our sample was representative of the overall adult population, we conducted annual capture events to compensate for mortality losses, and maintain balanced sex and age classes in our sample of marked individuals^79^. On average, 11% of study populations were marked and monitored each year (based on mark-resight aerial survey sightability estimation)^60^; a large proportion and overall sample size (*n* = 421 individuals) for deriving reliable estimates of avalanche-related survival^80^.

### Statistics and reproducibility

All statistical and summary analyses (described above) including visualizations were performed using Program R v4.3.1 and Microsoft Excel (v. 2019). Map visualizations were performed using ArcMap (v. 10.8).

### Reporting summary

Further information on research design is available in the Nature Portfolio Reporting Summary linked to this article.

### Supplementary information

Supplemental Material

Reporting Summary

## Data Availability

Data needed to reproduce the findings are publicly archived in the Dryad data repository^81^. Mountain goat location data are administered by the Alaska Department of Fish and Game, Division of Wildlife Conservation and are not freely available due to conservation concerns [Alaska Statute 16.05.815(d)] but may be requested by qualified parties through a data sharing agreement.

## Code availability

The code for the statistical analysis in the current study are available from the corresponding author on request.

## References

[1] Hock, R. et al. “High Mountain Areas” in IPCC Special Report on the Ocean and Cryosphere in a Changing Climate (Cambridge University Press, 2019). 10.1017/9781009157964.010

[2] Pepin, N. C. et al. Climate changes and their elevational patterns in the mountains of the world. *Rev. Geophys*. 60, 10.1029/2020RG000730 (2022).

[3] Parmesan C (2006). Ecological and evolutionary responses to recent climate change. Annu. Rev. Ecol. Evol. Syst..

[4] Natori Y, Porter WP (2007). Model of Japanese serow (*Capricornis crispus*) energetics predicts distribution on Honshu, Japan. Ecol. Appl..

[5] White KS, Gregovich DP, Levi T (2018). Projecting the future of an alpine ungulate under climate change scenarios. Glob. Change Biol..

[6] Lovari S (2020). Climatic changes and the fate of mountain herbivores. Clim. Change.

[7] Penczykowski RM, Connolly BM, Barton BT (2017). Winter is changing: trophic interactions under altered snow regimes. Food Webs.

[8] Boelman NT (2019). Integrating snow science and wildlife ecology in Arctic-boreal North America. Environ. Res. Lett..

[9] Dailey TV, Hobbs NT (1989). Travel in alpine terrain: energy expenditures for locomotion by mountain goats and bighorn sheep. Can. J. Zool..

[10] Jędrzejewski W, Jędrzejewska B, Okarma H, Ruprecht AL (1992). Wolf predation and snow cover as mortality factors in the ungulate community of the Bialowieża National Park, Poland. Oecologia.

[11] Pettorelli N, Pelletier F, von Hardenberg A, Festa-Bianchet M, Côté SD (2007). Early onset of vegetation growth vs. rapid green-up: impacts on juvenile mountain ungulates. Ecology.

[12] Berger J, Hartway C, Gruzdev A, Johnson M (2018). Climate degradation and extreme icing events constrain life in cold-adapted mammals. Sci. Rep..

[13] Sarmento W, Berger J (2020). Conservation implications of using an imitation carnivore to assess rarely used refuges as critical habitat features in an alpine ungulate. PeerJ.

[14] Jonas T, Geiger F, Jenny H (2008). Mortality pattern of the alpine chamois: the influence of snow–meteorological factors. Ann. Glaciol..

[15] White KS (2011). Mountain goat survival in coastal Alaska: effects of age, sex, and climate. J. Wildl. Manag..

[16] Conner MM (2018). Survival analysis: informing recovery of Sierra Nevada bighorn sheep. J. Wildl. Manag..

[17] Skladanowski MJ, Weidner EC, Bowles JL, Hebblewhite M (2021). Nineteen rocky mountain elk (*Cervus canadensis elson*) killed in an avalanche in the Three Sisters Wilderness. Northwest. Nat..

[18] Mainguy J, Côté SD, Festa-Bianchet M, Coltman DW (2009). Father–offspring phenotypic correlations suggest intralocus sexual conflict for a fitness-linked trait in a wild sexually dimorphic mammal. Proc. R. Soc. B..

[19] Schweizer J, Bruce Jamieson J, Schneebeli M (2003). Snow avalanche formation. Rev. Geophys..

[20] Hogarth RM, Lejarraga T, Soyer E (2015). The two settings of kind and wicked learning environments. Curr. Dir. Psychol. Sci..

[21] Fisher KC, Haegeli P, Mair P (2022). Exploring the avalanche bulletin as an avenue for continuing education by including learning interventions. J. Outdoor Recreat. Tour..

[22] Endler, J.A. *Natural Selection in the Wild*, Monographs in Population Biology (Princeton University Press, Princeton, N.J). 10.1126/science.233.4770.1332.a (1986). 10.1126/science.233.4770.1332 17843363

[23] Bühler Y (2018). Automated snow avalanche release area delineation – validation of existing algorithms and proposition of a new object-based approach for large-scale hazard indication mapping. Nat. Hazards Earth Syst. Sci..

[24] Christen M, Kowalski J, Bartelt P (2010). RAMMS: numerical simulation of dense snow avalanches in three-dimensional terrain. Cold Reg. Sci. Technol..

[25] Festa-Bianchet M, Coulson T, Gaillard J-M, Hogg JT, Pelletier F (2006). Stochastic predation events and population persistence in bighorn sheep. Proc. R. Soc. B.

[26] Hamel S, Côté SD, Smith KG, Festa-Bianchet M (2006). Population dynamics and harvest potential of mountain goat herds in Alberta. J. Wildl. Manag..

[27] Rice CG, Gay D (2010). Effects of mountain goat harvest on historic and contemporary populations. Northwest. Nat..

[28] White KS (2021). Integrating genetic data and demographic modeling to facilitate conservation of small, isolated mountain goat populations. J. Wildl. Manag..

[29] Jacobson AR, Provenzale A, Von Hardenberg A, Bassano B, Festa-Bianchet M (2004). Climate forcing and density dependence in a mountain ungulate population. Ecology.

[30] Rughetti M, Toïgo C, Von Hardenberg A, Rocchia E, Festa-Bianchet M (2011). Effects of an exceptionally snowy winter on chamois survival. Acta Theriol..

[31] DeCesare N, Smith B (2018). Contrasting native and introduced mountain goat populations in Montana.. Proc. North. Wild Sheep Goat Counc..

[32] White KS, Gregovich DP (2017). Mountain goat resource selection in relation to mining‐related disturbance. Wildl. Biol..

[33] McClung, D. & Schaerer, P. A. *The Avalanche Handbook* 3rd edn, (Mountaineers Books, Seattle, WA, 2006).

[34] Rixen C, Haag S, Kulakowski D, Bebi P (2007). Natural avalanche disturbance shapes plant diversity and species composition in subalpine forest belt. J. Veg. Sci..

[35] O’Leary D, Inouye D, Dubayah R, Huang C, Hurtt G (2020). Snowmelt velocity predicts vegetation green-wave velocity in mountainous ecological systems of North America. Int. J. Appl. Earth Obs..

[36] Hale R, Swearer SE (2016). Ecological traps: current evidence and future directions. Proc. R. Soc. B..

[37] Spitz DB, Hebblewhite M, Stephenson TR, German DW (2018). How plastic is migratory behavior? Quantifying elevational movement in a partially migratory alpine ungulate, the Sierra Nevada bighorn sheep (*Ovis canadensis sierrae*). Can. J. Zool..

[38] Lowrey, B. et al. Individual variation creates diverse migratory portfolios in native populations of a mountain ungulate. *Ecol. Appl*. 30 10.1002/eap.2106 (2020). 10.1002/eap.2106 32091631

[39] Berg JE, Hebblewhite M, St Clair CC, Merrill EH (2019). Prevalence and mechanisms of partial migration in ungulates. Front. Ecol. Evol..

[40] Peitzsch E, Pederson GT, Birkeland KW, Hendrikx J, Fagre DB (2021). Climate drivers of large magnitude snow avalanche years in the U.S. northern Rocky Mountains. Sci. Rep..

[41] Ballesteros-Cánovas JA, Trappmann D, Madrigal-González J, Eckert N, Stoffel M (2018). Climate warming enhances snow avalanche risk in the Western Himalayas. Proc. Natl. Acad. Sci. USA.

[42] Giacona F (2021). Upslope migration of snow avalanches in a warming climate. Proc. Natl. Acad. Sci. USA.

[43] Tabari H (2021). Extreme value analysis dilemma for climate change impact assessment on global flood and extreme precipitation. J. Hydrol..

[44] Beniston M, Stoffel M (2016). Rain-on-snow events, floods and climate change in the Alps: events may increase with warming up to 4 °C and decrease thereafter. Sci. Total Environ..

[45] Musselman KN (2018). Projected increases and shifts in rain-on-snow flood risk over western North America. Nat. Clim. Change.

[46] Hägeli P, McClung DM (2003). Avalanche characteristics of a transitional snow climate—Columbia Mountains, British Columbia, Canada. Cold Reg. Sci. Technol..

[47] Strapazzon G (2021). Effects of climate change on avalanche accidents and survival. Front. Physiol..

[48] Büntgen, U. et al. Elevational range shifts in four mountain ungulate species from the Swiss Alps. *Ecosphere*. 8 10.1002/ecs2.1761 (2017).

[49] Glazovskaya TG (1998). Global distribution of snow avalanches and changing activity in the Northern Hemisphere due to climate change. Ann. Glaciol..

[50] Shackleton, D. M. (ed.) *Wild sheep and goats and their relatives: status survey and conservation action plan for Caprinae* (IUCN, Gland, Switzerland, 1997).

[51] Wilson EE, Wolkovich EM (2011). Scavenging: how carnivores and carrion structure communities. Trends Ecol. Evol..

[52] Bell E, Fisher JT, Darimont C, Hart H, Bone C (2023). Influence of heterospecifics on mesocarnivore behaviour at shared scavenging opportunities in the Canadian Rocky Mountains. Sci. Rep..

[53] Rofkar T (2014). Managing and harvesting mountain goats for traditional purposes by indigenous user groups. Proc. North. Wild Sheep Goat Counc..

[54] Tepper, L. H. & George J., Joseph W. *Salish blankets: robes of protection and transformation, symbols of wealth,* (University of Nebraska Press, Lincoln, NB, 2017).

[55] Jessen TD (2022). Indigenous peoples as sentinels of change in human‐wildlife relationships: conservation status of mountain goats in Kitasoo Xai’xais territory and beyond. Conser. Sci. Pract..

[56] Gallant A. L., Binnian J. M., Omernik J. M. & Shasby M. B. *Ecoregions of Alaska,* (U.S. Government Printing Office, Washington D. C., 1995)

[57] Fellman JB (2014). Stream temperature response to variable glacier coverage in coastal watersheds of Southeast Alaska. Hydrol. Process..

[58] Shanley CS (2015). Climate change implications in the northern coastal temperate rainforest of North America. Clim. Change.

[59] Stowell H. H. *Geology of Southeast Alaska: Rock and Ice in Motion,* (University of Alaska Press, 2006).

[60] White K. S., Pendleton G. W. & Waite J. N. *Development of an aerial survey population estimation technique for mountain goats in Alaska*. (Final Wildlife Research Report ADF&G/DWC/WRR-2016-9, Alaska Department of Fish and Game, Division of Wildlife Conservation, Juneau, AK, 2016).

[61] Shafer ABA, Côté SD, Coltman DW (2011). Hot spots of genetic diversity descended from multiple Pleistocene refugia in an alpine ungulate. Evolution.

[62] Shafer ABA (2012). Habitat selection predicts genetic relatedness in an alpine ungulate. Ecology.

[63] White KS (2012). Modeling resource selection of mountain goats in southeastern Alaska: applications for population management and highway development planning. Proc. North. Wild Sheep Goat Counc..

[64] White K. S. & Gregovich D. P. “*Mountain goat resource selection in the Haines-Skagway area: Implications for helicopter skiing management*.” (Wildlife Research Report ADF&G/DWC/WRR–2018–2, Alaska Department of Fish and Game, Division of Wildlife Conservation, Juneau, AK., 2018).

[65] Shakeri YN, White KS, Waite JN (2021). Staying close to home: ecological constraints on space use and range fidelity in a mountain ungulate. Ecol. Evol..

[66] Smith CA, Raedeke KJ (1982). Group size and movements of a dispersed, low density goat population with comments on inbreeding and human impact. Proc. North. Wild Sheep Goat Counc..

[67] MacDonald SO, Cook JA (1996). The land mammal fauna of Southeast Alaska. Can. Field Nat..

[68] White KS, Watts DE, Beckmen KB (2021). Helicopter‐based chemical immobilization of mountain goats in coastal Alaska. Wildl. Soc. B..

[69] Smith BL (1988). Criteria for determining age and sex of American mountain goats in the field. J. Mammal..

[70] Brandborg S. M. Life history and management of the mountain goat in Idaho. *Idaho Wildlife Bull.* **2**, 1–142 (1955).

[71] Sikes RS (2016). the Animal Care and Use Committee of the American Society of Mammalogists, 2016 Guidelines of the American Society of Mammalogists for the use of wild mammals in research and education. J. Mammal..

[72] Bühler, Y. et al. Linking modelled potential release areas with avalanche dynamic simulations: an automated approach for efficient avalanche hazard indication mapping. *International Snow Science Workshop Proceedings*, 810–814. (Innsbruck, Austria, 2018).

[73] Lader R, Bidlack A, Walsh JE, Bhatt US, Bieniek PA (2020). Dynamical downscaling for Southeast Alaska: historical climate and future projections. J. Appl. Meterol. Clim..

[74] Bühler Y (2022). Automated avalanche hazard indication mapping on a statewide scale. Nat. Hazards Earth Syst. Sci..

[75] D’Eon RG, Serrouya R, Smith G, Kochanny C (2002). GPS radiotelemetry error and bias in mountainous terrain. Wildl. Soc. B..

[76] D’Eon RG, Delparte D (2005). Effects of radio-collar position and orientation on GPS radio-collar performance, and the implications of PDOP in data screening: Effects of radio-collar position. J. Appl. Ecol..

[77] Pollock KH, Winterstein SR, Bunck CM, Curtis PD (1989). Survival analysis in telemetry studies: the staggered entry design. J. Wildl. Manag..

[78] Wagler, B. L. et al. Effects of helicopter net‐gunning on survival of bighorn sheep. *J. Wildl. Manag*. 86 (2022), 10.1002/jwmg.22181.

[79] Prichard AK, Joly K, Dau J (2012). Quantifying telemetry collar bias when age is unknown: a simulation study with a long-lived ungulate. J. Wildl. Manag..

[80] Murray DL (2006). On improving telemetry-based survival estimation. J. Wildl. Manag..

[81] White, K. et al. Long-term individual-based records of mountain goat mortality and terrain use in relation to avalanches in coastal Alaska during 2005-2022. [Dataset]. Dryad. 10.5061/dryad.xsj3tx9ms (2024).

