# Supplementary material for: Snow avalanches are a primary climate-linked driver of mountain ungulate populations
Source: Commun Biol. 2024 Apr 29;7:423. doi: 10.1038/s42003-024-06073-0 (PMC11058775; doi:10.1038/s42003-024-06073-0)
Supplement: Supplementary file 1 — Supplemental Material [file 42003_2024_6073_MOESM1_ESM.docx]

**Supplementary information for:**

**Snow avalanches are a primary climate-linked driver of mountain ungulate populations**

Kevin S. White^1,2,3,*^, Eran Hood^1^, Gabriel J. Wolken^4,5^, Erich H. Peitzsch^6^, Yves Bühler^7,8^, Katreen Wikstrom Jones^5^, and Chris T. Darimont^2^

**Affiliations:**

^1^Program on the Environment, Department of Natural Sciences, University of Alaska Southeast; Juneau, AK 99801, USA.

^2^Department of Geography, University of Victoria; Victoria, BC V8W 2Y2, Canada.

^3^Division of Wildlife Conservation, Alaska Department of Fish and Game; Juneau, AK 99811, USA (Ret.).

^4^Climate and Cryosphere Hazards Program, Alaska Division of Geological and Geophysical Surveys; Fairbanks, AK 99709, USA.

^5^Alaska Climate Adaptation Science Center, University of Alaska Fairbanks; Fairbanks, AK 99775, USA.

^6^U.S. Geological Survey, Northern Rocky Mountain Science Center; West Glacier, MT 59936, USA.

^7^WSL Institute for Snow and Avalanche Research SLF, Davos CH-7260, Switzerland.

^8^Climate Change, Extremes and Natural Hazards in Alpine Regions Research Centre CERC, Davos CH-7260, Switzerland.

***Corresponding author:** Kevin White

**E-mail:** [kwhite27@alaska.edu](mailto:kwhite27@alaska.edu)


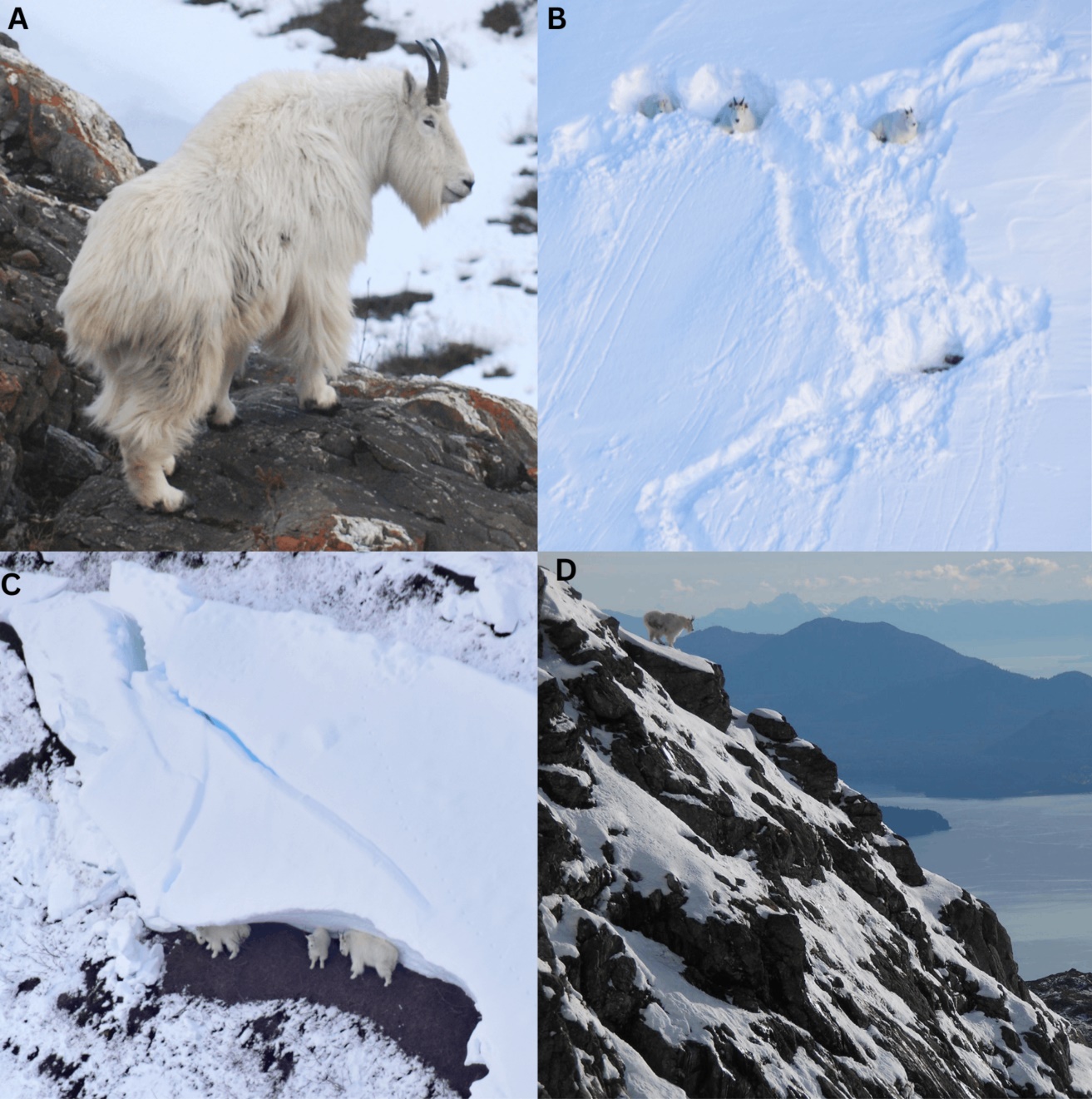


**Supplementary Figure 1. Mountain goats and avalanches.** **(A)** Adult male mountain goat in late-winter illustrating specialized adaptations for mountain environments including thick, woolen coat, muscular shoulders, narrow body width and hooves with hard keratinous sheaths and soft, adhesive interior pads (not seen). **(B)** Mountain goats in extensively excavated beds within mapped avalanche terrain following a major snowfall event (2.4 m over 6 days) during December 2020, Porcupine Mountain, Klukwan, Alaska. **(C)** Mountain goats sheltering beneath the fracture line of a mid-winter glide avalanche, Summit Creek, Klukwan, Alaska. **(D)** Adult female mountain goat navigating a 40° slope, Lions Head Mountain, Lynn Canal, Alaska. Resource selection function modeling indicates mountain goats optimally select for slope angles (36-58°) that coincide with those at which avalanches are most likely to release in maritime snow climates (30-45°).


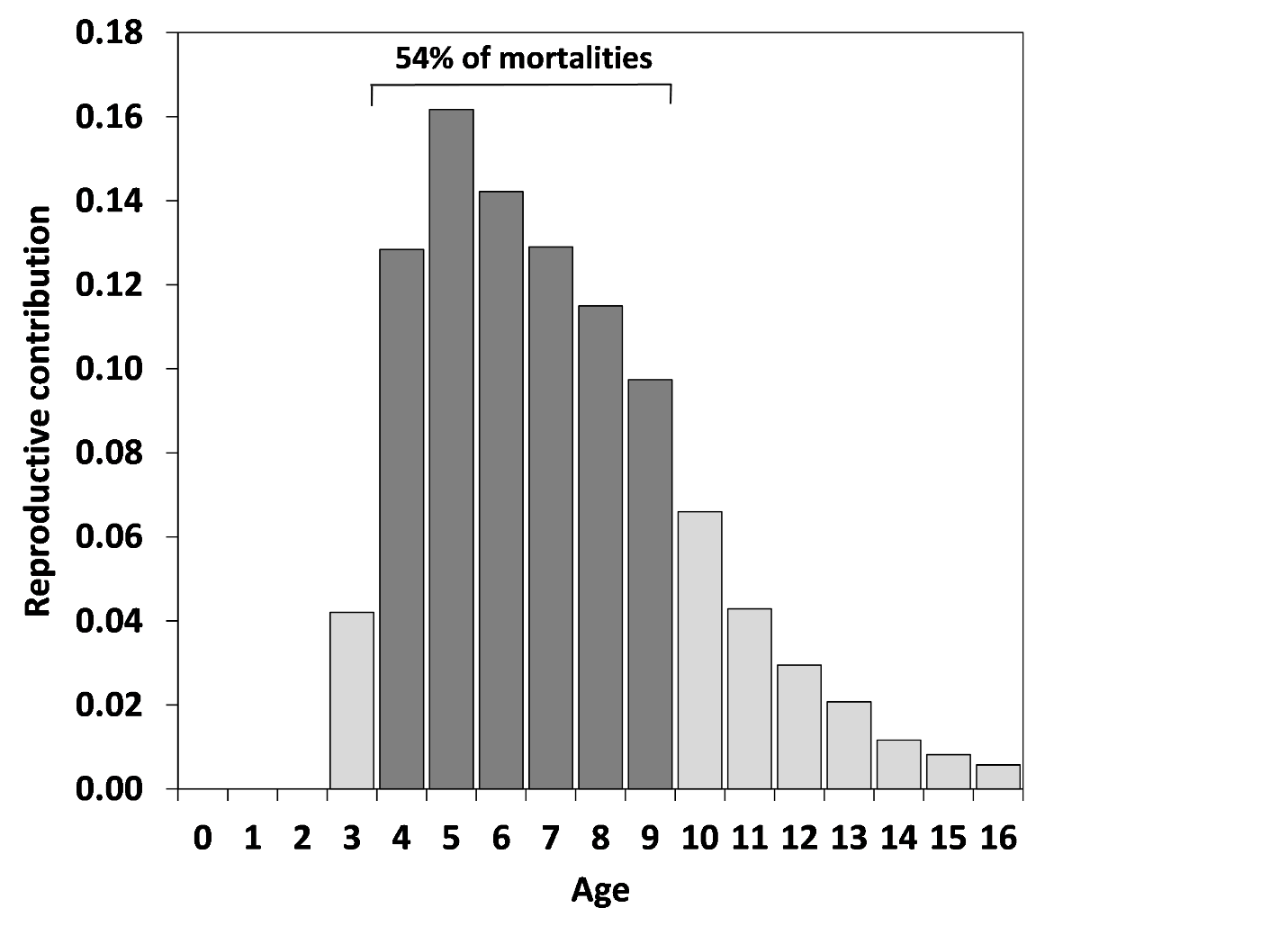


**Supplementary Figure 2. Avalanche impacts on prime-aged females.** Avalanches exert strong effects on prime, reproductive-aged females, as illustrated by the relationship between age-specific female mountain goat reproductive contribution in relation avalanche-caused mortality. Among female mountain goats that died in avalanches, most occur among individuals in prime-aged classes (54%). Adult females in prime-aged classes contribute 77% of new recruits into a population annually. Prime aged (4-9 yrs) is defined as the population component with the highest age-specific fecundity and survival (White et al. 2011, 2018). Population age structure was calculated based on average population conditions for mountain goats in southeastern Alaska (White et al. 2021). Age-specific reproductive contribution was calculated by multiplying by age-specific fecundity by the female stable age distribution (White et al. 2018, 2021).


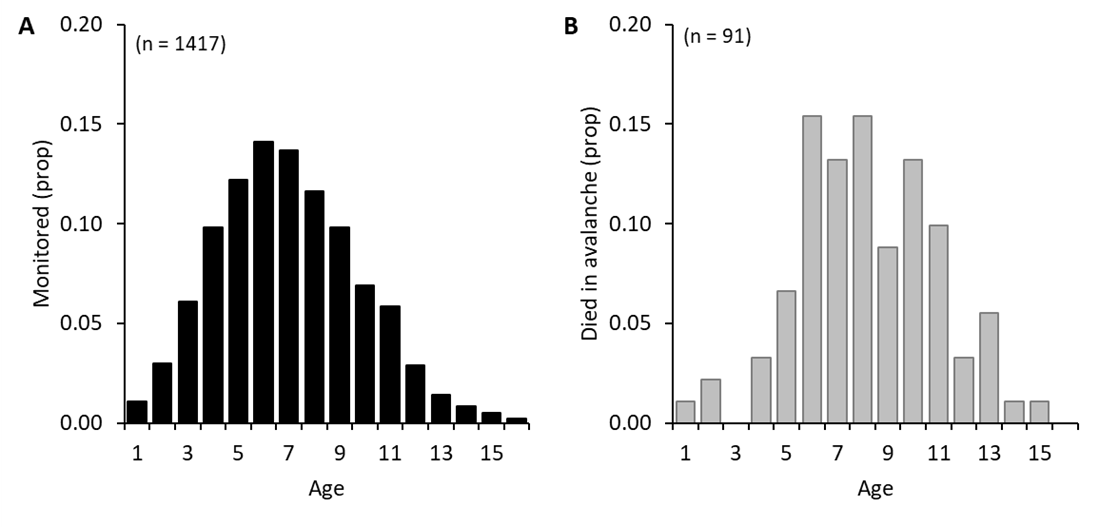


**Supplementary Figure 3. Age distribution of radio-marked mountain goats. (A)** The proportion of radio-marked mountain goats monitored in each age class. **(B)** The proportion of radio-marked mountain goats that died in avalanches in each age class. Radio-marked mountain goats were monitored in four separate populations in coastal Alaska during 2005-2022. It was not possible to precisely ascertain age in two instances.


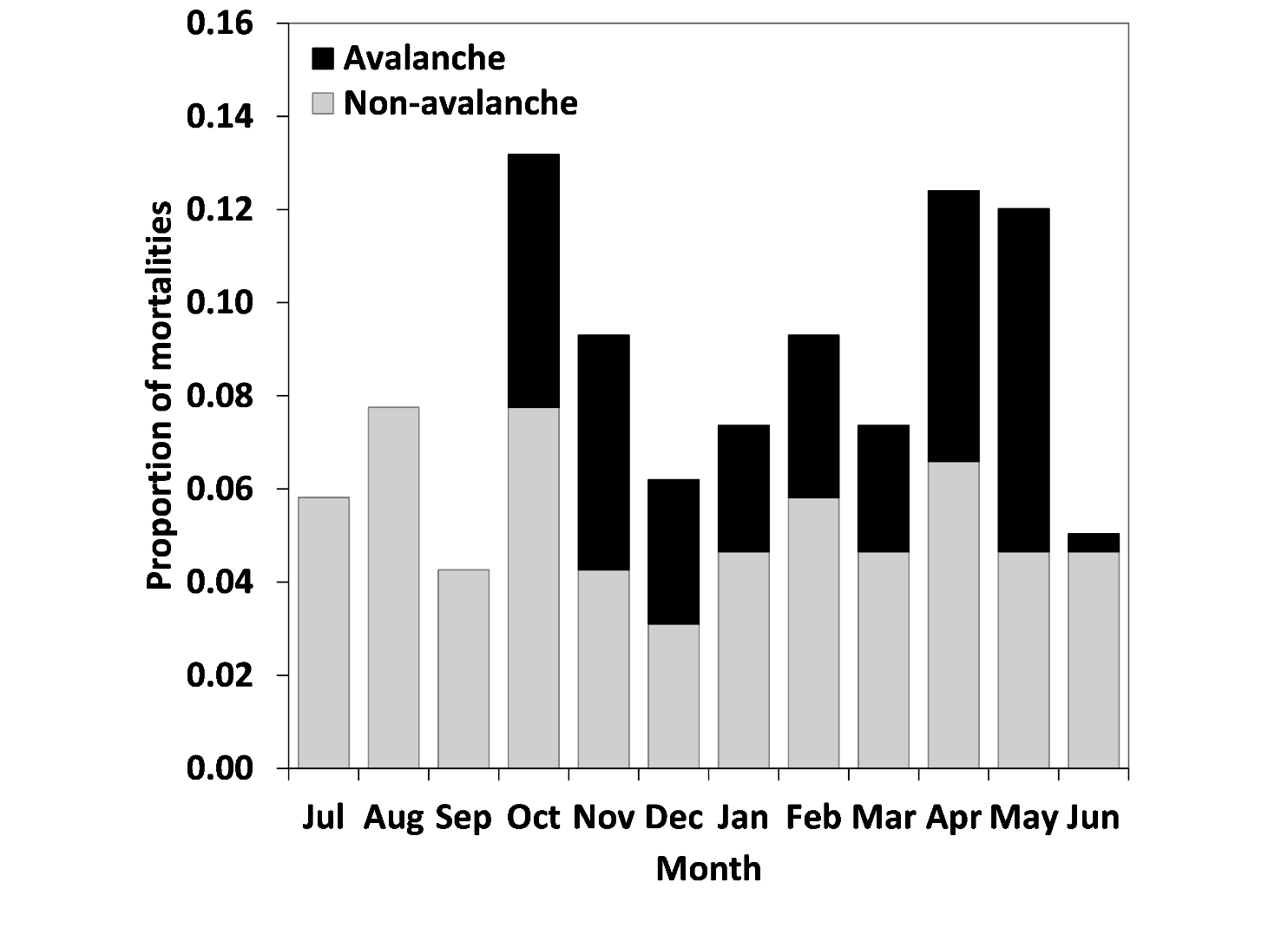


**Supplementary Figure 4.** **Temporal patterns of mountain goat mortality.** Avalanche-caused mortalities of radio-marked mountain goats (n = 93) occurred during nine months, and peaked in early-autumn and late-spring in coastal Alaska (2005-2022). Non-avalanche related mortalities (n = 165) occurred consistently throughout the year.


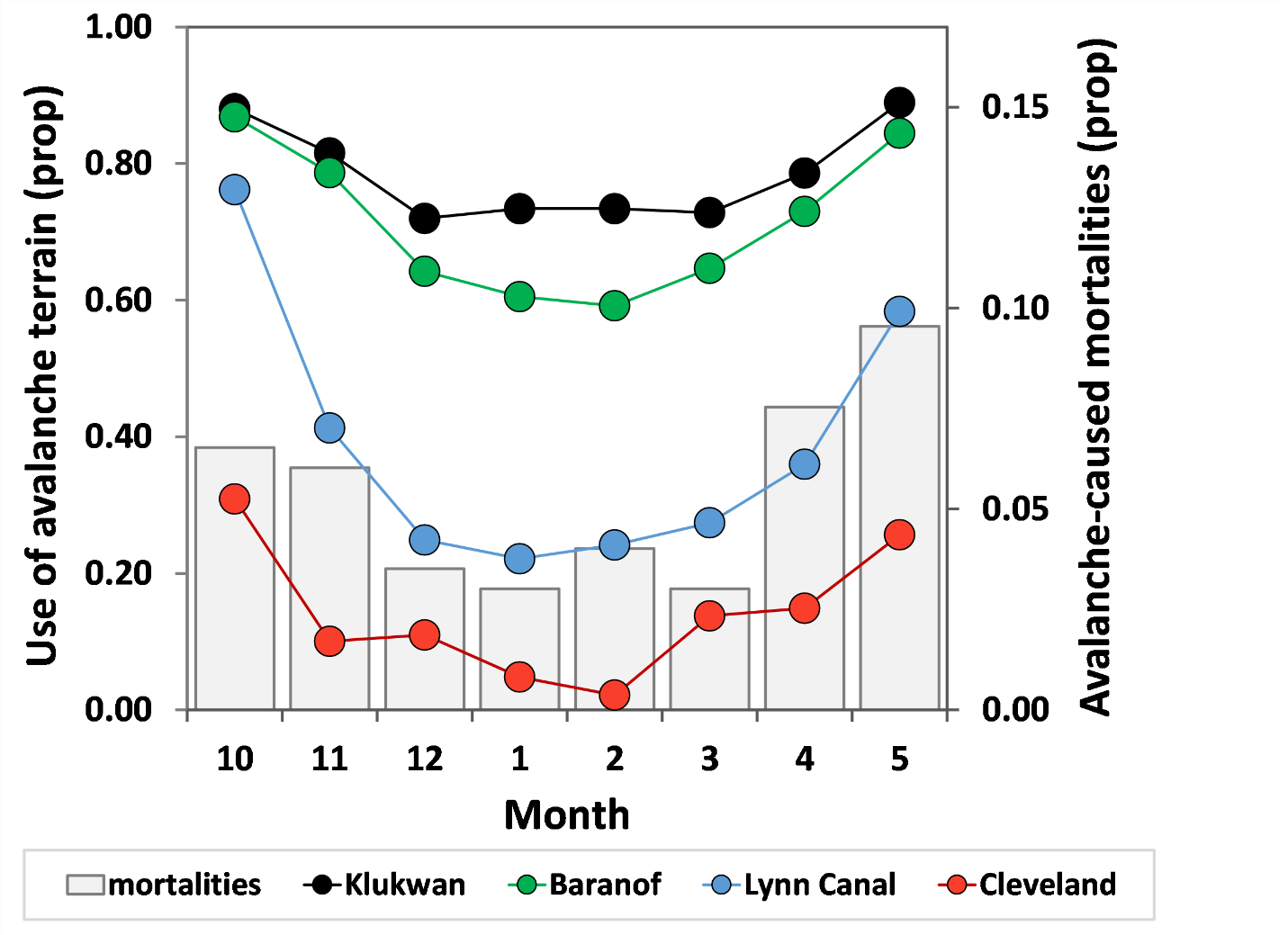


**Supplementary Figure 5. Mountain goat use of avalanche terrain.** Spatial and temporal variation in the use of avalanche terrain by mountain goats in four study areas (color coded, left y-axis) in southeastern Alaska during 2005 – 2022. Proportion of mortalities caused by avalanches is summarized by month for the period with snow (grey bars, secondary y-axis) and includes all GPS plus VHF radio-monitored animals (total avalanche mortalities, n = 93). Avalanche terrain includes non-forested predicted avalanche release areas and avalanche paths derived using the RAMMS avalanche simulation model.


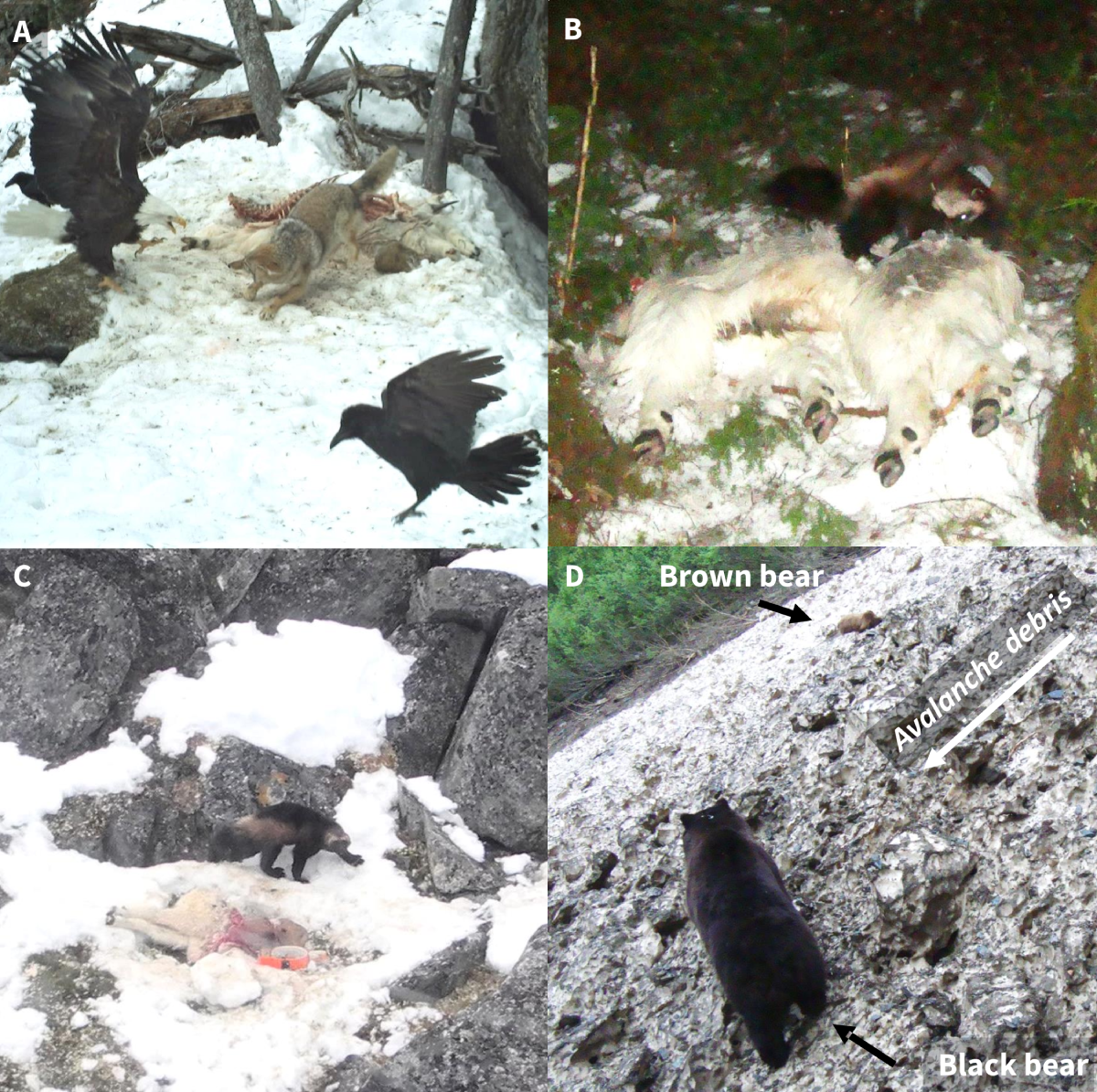


**Supplementary Figure 6. Mountain goat carcass subsidy of scavenger food webs.** Mountain goat carrion represents an important, and contested, food resource among mountain scavengers that commonly occur in avalanche terrain, and also other habitat types. **(A)** Coyote (*Canis latrans*) defending a mountain goat carcass from a bald eagle (*Haliaeetus leucocephalus*), with a raven (*Corvus corax*) in association, Klukwan, Alaska. **(B)** Wolverine (*Gulo gulo*) scavenging on a mountain goat in low elevation forested habitat during late-winter, Lynn Canal, Alaska. **(C)** Wolverine scavenging on a mountain goat carcass in high elevation alpine habitat during autumn, Lynn Canal, Alaska. **(D)** Brown bear (*Ursus arctos*) excavating a mountain goat carcass from an avalanche chute, while a black bear (*Ursus americanus*) observes from distance, Klukwan, Alaska.

**Supplementary Table 1. Avalanche footprint in Alaska Coast Range mountains.** Area and percentage of avalanche terrain (including release areas and avalanche paths) within each of the four coastal Alaska mountain goat study areas, Alaska.

|  | Area (km^2^) | | |  |
| --- | --- | --- | --- | --- |
| Study Area | Non-avalanche | Avalanche | Total | Avalanche (prop.) |
| Klukwan | 1064 | 2148 | 3212 | 0.67 |
| Baranof | 369 | 572 | 941 | 0.61 |
| Lynn Canal | 534 | 697 | 1231 | 0.57 |
| Cleveland Pen. | 142 | 12 | 153 | 0.08 |
| Total | 2108 | 3429 | 5537 | 0.62 |

**Supplementary Table 2. Mountain goat use of avalanche terrain and survival.** GPS radio-collared mountain goat use of avalanche terrain during winter months in relation to whether death occurred by avalanche. Data were collected in four study areas in southeastern Alaska during 2005-2022. Avalanche terrain includes non-forested predicted avalanche release areas and avalanche paths derived using the RAMMS avalanche simulation model. Mountain goat GPS radio-collar location data were collected from a subset (87%) of the total individuals monitored (n = 421).

|  | Avalanche mortality: Yes | | |  | Avalanche mortality: No | | |  |  |  |
| --- | --- | --- | --- | --- | --- | --- | --- | --- | --- | --- |
| Month | mean | SE | n |  | mean | SE | n |  | t-value | P-value |
| Oct | 0.85 | 0.02 | 85 |  | 0.80 | 0.01 | 282 |  | 1.847 | 0.03 |
| Nov | 0.70 | 0.03 | 85 |  | 0.58 | 0.02 | 274 |  | 3.528 | <0.01 |
| Dec | 0.56 | 0.04 | 81 |  | 0.44 | 0.02 | 272 |  | 2.813 | <0.01 |
| Jan | 0.56 | 0.04 | 81 |  | 0.41 | 0.02 | 268 |  | 3.570 | <0.01 |
| Feb | 0.55 | 0.04 | 79 |  | 0.42 | 0.02 | 265 |  | 3.181 | <0.01 |
| Mar | 0.59 | 0.04 | 75 |  | 0.44 | 0.02 | 261 |  | 3.244 | <0.01 |
| Apr | 0.65 | 0.04 | 71 |  | 0.53 | 0.02 | 252 |  | 2.713 | <0.01 |
| May | 0.78 | 0.03 | 62 |  | 0.70 | 0.02 | 237 |  | 1.782 | 0.04 |
| All Months | 0.67 | 0.03 | 85 |  | 0.54 | 0.02 | 282 |  | 3.643 | <0.01 |

**Supplementary Table 3: Causes of mountain goat mortality**. Summary of fates among radio-marked mountain goats that died (n = 258) in four study areas in southeastern Alaska during 2005 – 2022. Causes of mortality were discerned during aerial reconnaissance and follow-up field investigations. Accidental causes were clearly definitive in all instances. Cases of malnutrition involved definitive proximate evidence of nutritional deprivation. Unknown causes of mortality occurred when evidence of large carnivores, that both kill and scavenge mountain goats, was present but not definitively linked to predation; such cases were considered to represent instances of either malnutrition or predation-related mortality. It is not possible to determine the faction of unknown mortalities caused by predation vs malnutrition based on identified proportions of non-accident causes of mortality.

| Cause | Cases | Proportion |
| --- | --- | --- |
| Accident |  |  |
| Avalanche | 93 | 36% |
| Fall | 6 | 2% |
| Subtotal | 99 | 38% |
|  |  |  |
| Non-accident |  |  |
| Malnutrition | 19 | 7% |
| Predation (Wolf) | 19 | 7% |
| Predation (Bear - Brown or Black) | 16 | 6% |
| Predation (Unknown Type) | 5 | 2% |
| Unknown (Non-accident) | 100 | 39% |
| Subtotal | 159 | 62% |
| Total | 258 | 100% |
