# Supplementary material for: Snow avalanches are a primary climate-linked driver of mountain ungulate populations
Source: Commun Biol. 2024 Apr 29;7:423. doi: 10.1038/s42003-024-06073-0 (PMC11058775; doi:10.1038/s42003-024-06073-0)
Supplement: Supplementary file 2 — Reporting Summary [file 42003_2024_6073_MOESM2_ESM.pdf]

nature portfolio

Corresponding author(s): Kevin White

Last updated by author(s): Feb 28, 2024

Reporting Summary

Nature Portfolio wishes to improve the reproducibility of the work that we publish. This form provides structure for consistency and transparency in reporting. For further information on Nature Portfolio policies, see our Editorial Policies and the Editorial Policy Checklist.

Statistics

For all statistical analyses, confirm that the following items are present in the figure legend, table legend, main text, or Methods section.

n/a	Confirmed	
☐	☒	The exact sample size (*n*) for each experimental group/condition, given as a discrete number and unit of measurement
☐	☒	A statement on whether measurements were taken from distinct samples or whether the same sample was measured repeatedly
☐	☒	The statistical test(s) used AND whether they are one- or two-sided<br>*Only common tests should be described solely by name; describe more complex techniques in the Methods section.*
☐	☒	A description of all covariates tested
☐	☒	A description of any assumptions or corrections, such as tests of normality and adjustment for multiple comparisons
☐	☒	A full description of the statistical parameters including central tendency (e.g. means) or other basic estimates (e.g. regression coefficient) AND variation (e.g. standard deviation) or associated estimates of uncertainty (e.g. confidence intervals)
☐	☒	For null hypothesis testing, the test statistic (e.g. F, t, r) with confidence intervals, effect sizes, degrees of freedom and P value noted<br>*Give P values as exact values whenever suitable.*
☒	☐	For Bayesian analysis, information on the choice of priors and Markov chain Monte Carlo settings
☒	☐	For hierarchical and complex designs, identification of the appropriate level for tests and full reporting of outcomes
☒	☐	Estimates of effect sizes (e.g. Cohen's *d*, Pearson's *r*), indicating how they were calculated

Our web collection on statistics for biologists contains articles on many of the points above.

Software and code

Policy information about availability of computer code

Data collection	No software was used to collect data
Data analysis	We conducted standard statistical analyses and data summaries using Program R and Microsoft Excel. We used numerical dynamic avalanche model Rapid Mass Movement Simulations (RAMMS) to spatially delineate avalanche terrain.

For manuscripts utilizing custom algorithms or software that are central to the research but not yet described in published literature, software must be made available to editors and reviewers. We strongly encourage code deposition in a community repository (e.g. GitHub). See the Nature Portfolio guidelines for submitting code & software for further information.

Data

Policy information about availability of data

All manuscripts must include a data availability statement. This statement should provide the following information, where applicable:

- Accession codes, unique identifiers, or web links for publicly available datasets
- A description of any restrictions on data availability
- For clinical datasets or third party data, please ensure that the statement adheres to our policy

Data needed to reproduce the findings are described in the supplementary materials and publicly archived in the Dryad data repository (https://doi.org/10.5061/dryad.xsj3tx9ms). Mountain goat location data are administered by the Alaska Department of Fish and Game, Division of Wildlife Conservation and are not freely available due to conservation concerns [Alaska Statute 16.05.815(d)] but may be requested by qualified parties through a data sharing agreement.

Research involving human participants, their data, or biological material

Policy information about studies with human participants or human data. See also policy information about sex, gender (identity/presentation), and sexual orientation and race, ethnicity and racism.

Reporting on sex and gender	n/a
Reporting on race, ethnicity, or other socially relevant groupings	n/a
Population characteristics	n/a
Recruitment	n/a
Ethics oversight	n/a

Note that full information on the approval of the study protocol must also be provided in the manuscript.

Field-specific reporting

Please select the one below that is the best fit for your research. If you are not sure, read the appropriate sections before making your selection.

☐ Life sciences ☐ Behavioural & social sciences ☒ Ecological, evolutionary & environmental sciences

For a reference copy of the document with all sections, see nature.com/documents/nr-reporting-summary-flat.pdf

Ecological, evolutionary & environmental sciences study design

All studies must disclose on these points even when the disclosure is negative.

Study description	We conducted a field study focused on monitoring spatial and temporal patterns of mortality among individually marked mountain goats, in relation to avalanche risk. Mountain goats were captured using standard methodology, deployed with radio-collars (VHF/ GPS), and systematically monitored on a monthly basis. When mortalities were detected, sites were visited to ascertain causes of death. GPS radio-collar location data were intersected with spatially-explicit avalanche terrain models and spatial use patterns were contrasted between individuals that died from avalanches and those that died from other causes.
Research sample	Sampling units were comprised of individual mountain goats (Oreamnos americanus), and included all sex- and age-classes (except neonates). Sampling occurred in four study areas (Lynn Canal, Klukwan, Baranof Island, cleveland Peninsula) located in southeastern Alaska. The sample spanned a broad range of geographic and climatic conditions in the region, and is considered to be representative of coastal mountain goat populations.
Sampling strategy	Individual adult mountain goats were selected for capture and radio-marking within each study area using a stratified, random design. That is, animals were randomly selected for capture within each discrete geographic area. Overall, we monitored 421 individuals in four separate study areas across a 17 year period. The sampling effort is among the most extensive ever conducted for this species and considered sufficient to rigorously address study questions.
Data collection	Individuals were then systematically monitored (monthly) during the study period. All deaths were investigated and carefully examined to determine cause of death at each mortality site. Kevin White and field staff (in acknowledgements) collected all field data.
Timing and spatial scale	Individual radio-marked mountain goats were monitored at monthly (or more frequent) intervals during 2005-2022 in four study areas located in southeastern Alaska (see below for a more detailed biogeographic description).
Data exclusions	Individual mountain goats that died from human harvest (hunting) were excluded from analyses because our study was focused on investigating only natural sources of mortality. This decision was made prior to data collection.
Reproducibility	We considered our large, spatially extensive sample size (421 individuals in four separate study areas across a 17 year period) to be representative of our study system and associated ecological dynamics.
Randomization	Individual adult mountain goats were selected for capture and radio-marking within each study area using a stratified, random design. That is, animals were randomly selected for capture within each discrete geographic area.
Blinding	Our study did not involve controlled, manipulative experiments and, thus, did not involve blinding.

Did the study involve field work? ☒ Yes ☐ No

Field work, collection and transport

Field conditions	Field conditions were typical of northern maritime mountain environments of coastal southeastern Alaska. Mean monthly temperatures range from -2°C to 14°C and mean annual precipitation is 1400 mm in Juneau. Across the region, annual precipitation ranges from 1 to >8 m and winter snowfall ranges from 0.5 to > 3 m of water equivalent. During the study period, annual snowfall at sea level in Juneau averaged 233 cm with a range of 89-501 cm.
Location	Mountain goats were studied in four separate areas across a broad geographic range in coastal Alaska (5537 km2; Lat: 55-59N, Long: -136-132W). This area is within the Pacific Coastal Mountains biogeographic region. The area is composed primarily of Sitka spruce-western hemlock (Picea sitchensis-Tsuga heterophylla) forests at lower elevations (below 450-750 m a.s.l.). At higher elevations, subalpine and alpine habitats dominated by krummholtz forest, low-growing herbaceous meadows and ericaceous heathlands are widespread and persist to elevations of about 1400 m a.s.l. The geologic terrain is complex and strongly influenced by terrain accretion and uplift processes resulting in a highly fractured landscape dominated by steep and rugged topography that is fragmented by active glaciers, icefields, high-volume river systems and marine waters. Glacier recession has heavily modified the region leaving steep, sloping topography. The avalanche paths in this study extend from sea level to 2000 m above sea level (a.s.l.) and include a variety of aspects as a result of the complex topography of the Coast Mountains.
Access & import/export	The study sites and subjects were accessed using aircraft (fixed-wing and helicopter), boats or ground-based means. Permits were not required to access field sites. Mountain goat capture and handling procedures employed standard techniques, and were reviewed and approved by the Alaska Department of Fish and Game Institutional Animal Care and Use Committee (protocols 05-11, 2016-25, 0078-2018-68, 0039-2017-39) and followed American Society of Mammalogists guidelines.
Disturbance	Mountain goats were disturbed during the initial capture process. Effects were short-term (30-45 minutes), carefully mitigated to the extent possible, and entailed strict adherence to animal welfare and ethics guidelines as required by the Alaska Department of Fish and Game Institutional Animal Care and Use Committee (protocols 05-11, 2016-25, 0078-2018-68, 0039-2017-39).

Reporting for specific materials, systems and methods

We require information from authors about some types of materials, experimental systems and methods used in many studies. Here, indicate whether each material, system or method listed is relevant to your study. If you are not sure if a list item applies to your research, read the appropriate section before selecting a response.

Materials & experimental systems

n/a	Involved in the study	
☒	☐	Antibodies
☒	☐	Eukaryotic cell lines
☒	☐	Palaeontology and archaeology
☐	☒	Animals and other organisms
☒	☐	Clinical data
☒	☐	Dual use research of concern
☒	☐	Plants

Methods

n/a	Involved in the study	
☒	☐	ChIP-seq
☒	☐	Flow cytometry
☒	☐	MRI-based neuroimaging

Animals and other research organisms

Policy information about studies involving animals; ARRIVE guidelines recommended for reporting animal research, and Sex and Gender in Research

Laboratory animals	The study did not involve laboratory animals.
Wild animals	The study involved field capture of wild animals (mountain goats). Capture methodology and results were previously described in detail (White et al. 2021. Helicopter-based chemical immobilization of mountain goats in coastal Alaska. Wildlife Society Bulletin 45, 670-681). Mountain goat capture and handling procedures employed standard techniques, and were reviewed and approved by the Alaska Department of Fish and Game Institutional Animal Care and Use Committee (protocols 05-11, 2016-25, 0078-2018-68, 0039-2017-39) and followed American Society of Mammalogists guidelines. Specifically, mountain goats were captured using standard helicopter darting methods and immobilized using carfentanil or thiafentanil via a projectile syringe; following handling, effects were reversed with naltrexone hydrochloride. During handling, standard veterinary procedures were used to ensure animal health and safety. Sex- and age-status was determined, and radio-collars (VHF/GPS) were fastened to each individual. Radio-collar batteries had a set life-span, and GPS collars were pre-programmed to release prior to end of battery life. During follow-up monitoring, individuals were detected and/or observed from fixed-wing aircraft (or via examination of GPS radio-collar data) to determine fates, including specific time and location of death (when death occurred). In cases of mortality, individual animal remains were examined to ascertain cause of death.
Reporting on sex	Adult male and female mountain goats were captured and monitored during this study. All individuals were captured, handled and monitored in an identical fashion, irrespective of sex status.

Field-collected samples	Field collected samples from live animals were not used in this study.
Ethics oversight	Mountain goat capture and handling procedures employed standard techniques, and complied with all relevant ethical regulations for animal use and were approved by the Alaska Department of Fish and Game Institutional Animal Care and Use Committee (protocols 05-11, 2016-25, 0078-2018-68, 0039-2017-39) and followed American Society of Mammalogists guidelines

Note that full information on the approval of the study protocol must also be provided in the manuscript.

Plants

Seed stocks	Plant samples were collected during this study.
Novel plant genotypes	n/a
Authentication	n/a
